# An autonucleolytic suspension HEK293F host cell line for high-titer serum-free AAV5 and AAV9 production with reduced levels of DNA impurity

**DOI:** 10.1016/j.omtm.2024.101317

**Published:** 2024-08-12

**Authors:** Geoffrey Howe, Mehtap Bal, Matt Wasmuth, Giulia Massaro, Ahad A. Rahim, Sadfer Ali, Milena Rivera, Desmond M. Schofield, Aminat Omotosho, John Ward, Eli Keshavarz-Moore, Chris Mason, Darren N. Nesbeth

**Affiliations:** 1 The Advanced Centre for Biochemical Engineering, Department of Biochemical Engineering, University College London, London WC1E 6BT, UK; 2 UCL School of Pharmacy, University College London, London WC1N 1AX, UK

**Keywords:** synthetic biology, gene therapy, autonucleolytic, AAV, cell engineering, DNA, bioprinting, scaffold

## Abstract

We sought to engineer mammalian cells to secrete nuclease activity as a step toward removing the need to purchase commercial nucleases as process additions in bioprocessing of AAV5 and AAV9 as gene therapy vectors. Engineering HeLa cells with a serratial nuclease transgene did not bring about nuclease activity in surrounding media whereas engineering serum-free, suspension-adapted HEK293F cells with a staphylococcal nuclease transgene did result in detectable nuclease activity in surrounding media of the resultant stable transfectant cell line, “NuPro-1S.” When cultivated in serum-free media, NuPro-1S cells yielded 3.06 × 10^10^ AAV5 viral genomes (vg)/mL via transient transfection, compared with 3.85 × 10^9^ vg/mL from the parental HEK293F cell line. AAV9 production, followed by purification by ultracentrifugation, yielded 1.8 × 10^13^ vg/mL from NuPro-1S cells compared with 7.35 × 10^12^ vg/mL from HEK293F cells. AAV9 from both HEK293F and NuPro-1S showed almost identical ability to transduce cells embedded in a scaffold tissue mimic or cells of mouse neonate brain tissue *in vivo*. Comparison of agarose gel data indicated that the DNA content of AAV5 and AAV9 process streams from NuPro-1S cells was reduced by approximately 60% compared with HEK293F cells. A similar reduction in HEK293F cells was only achievable with a 50 U/mL Benzonase treatment.

## Graphical abstract

**Figure:**
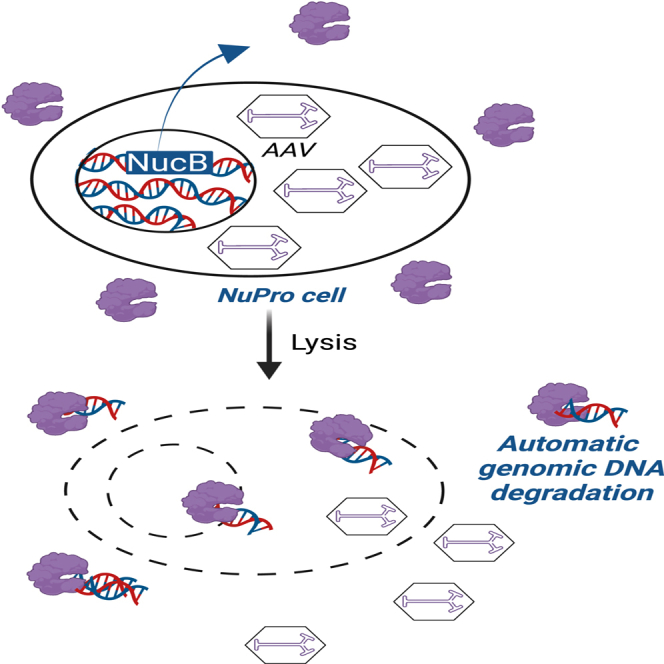

## Teaser

Nesbeth and colleagues engineered HEK293F cells with a transgene encoding a secreted staphylococcal nuclease. This engineering step did not compromise AAV5 or AVV9 yield or infectiousness when the cells were used as hosts for transient transfection, but did effect reduced levels of DNA impurity in process streams.

## Introduction

Recombinant adeno-associated viral vector (AAV) is predominantly generated by transient, triple plasmid transfection of adherent HEK293 cells. Proteins required for viral vector components and their assembly and production are encoded across two plasmids, the RepCap and Helper plasmids. A third plasmid encodes the recombinant payload genome.^1^ The cost of manufacturing AAV at scale remains a significant barrier to accessing gene therapy solutions for patient populations worldwide. For example, AAV-based gene therapies can currently range in price from $850,000 to $3 million.^2^^,^^3^

Adherent mammalian cells are always limited by the planar surface area of the horizontal vessels used for their cultivation. Cultivating adherent cells on the surface of microcarriers can improve the number of cells grown per unit volume but comes with additional costs of the microcarriers in addition to specialized vessel costs.^4^ Transient transfection of suspension cells has been increasingly trialed and adopted for large-scale AAV production, at scales up to 1,000 L.^5^^,^^6^^,^^7^^,^^8^^,^^9^^,^^10^^,^^11^^,^^12^

Artificial intelligence and automation are increasingly being applied to bioprocessing to help drive down costs and boost the yield of large-scale processes, and to enable distributed, small-scale manufacturing systems.^13^^,^^14^ “Factory in a box” (FIAB) approaches to gene therapy bioprocessing involve closed material handling and automated systems controlled algorithmically. For FIAB systems to flourish, bioprocesses must be simplified. Animal-derived materials, such as serum, that introduce batch variability and routes to contamination,^15^ must ideally be replaced with serum-free alternatives. Also, process additions, such as enzymes for nucleic acid clearance, must ideally be minimized to simplify and streamline unit operations.

The presence of DNA impurities in AAV material can trigger immune responses in humans.^16^ Consequently, the US Food and Drug Administration guidelines recommend that no more than 10 ng of DNA impurity is present in each dose of a gene therapy product and that any DNA impurity present is maximum 200 base pairs (bp) in length.^17^^,^^18^ To meet these requirements typically recombinant nucleases, such as Benzonase, are added to AAV process streams, at significant cost.^19^^,^^20^ DNA impurities in cell lysates have been observed to favor aggregation phenomena across a range of process streams, from bacteria for biologics production to mammalian cell-based AAV production,^21^^,^^22^ wherein aggregates can negatively impact downstream filtration performance.^23^

The *Staphylococcal aureus* nuclease, nucB (UniProt: P00644), has high levels of extracellular activity and heat stability for DNA and RNA hydrolysis.^24^ Wild-type and industrial *Escherichia coli* strains have previously been engineered to express nucB in their periplasmic space to safeguard cytoplasmic nucleic acids during cell growth.^25^^,^^26^ Upon homogenization to release a given product, nucB can then be liberated from the periplasm to access and hydrolyze nucleic acids.

Here, we attempt to engineer mammalian cells to secrete either *Serratia marcescens* nuclease (GenBank M19495) or *S. aureus* nucB. Native *S. marcescens* nuclease (abbreviated here as “SMnuc”) is the basis for the widely utilized Benzonase commercial nuclease product.^27^ We then test whether the nuclease-engineered status of host cells compromises their yield performance for production of recombinant AAV5 and AAV9 by three-plasmid transient transfection. We characterize the viral progeny of both unmodified and nuclease-engineered host cells with respect to their ability to transduce target cells on tissue culture well plates and embedded in a scaffold tissue mimic. We also test their ability to transduce cells of mouse brain tissue *in vivo*. Finally, we test the stability of the phenotype that leads to medium-resident nuclease activity over 31 passages performed over 3 months.

## Results

**Figure 1:**
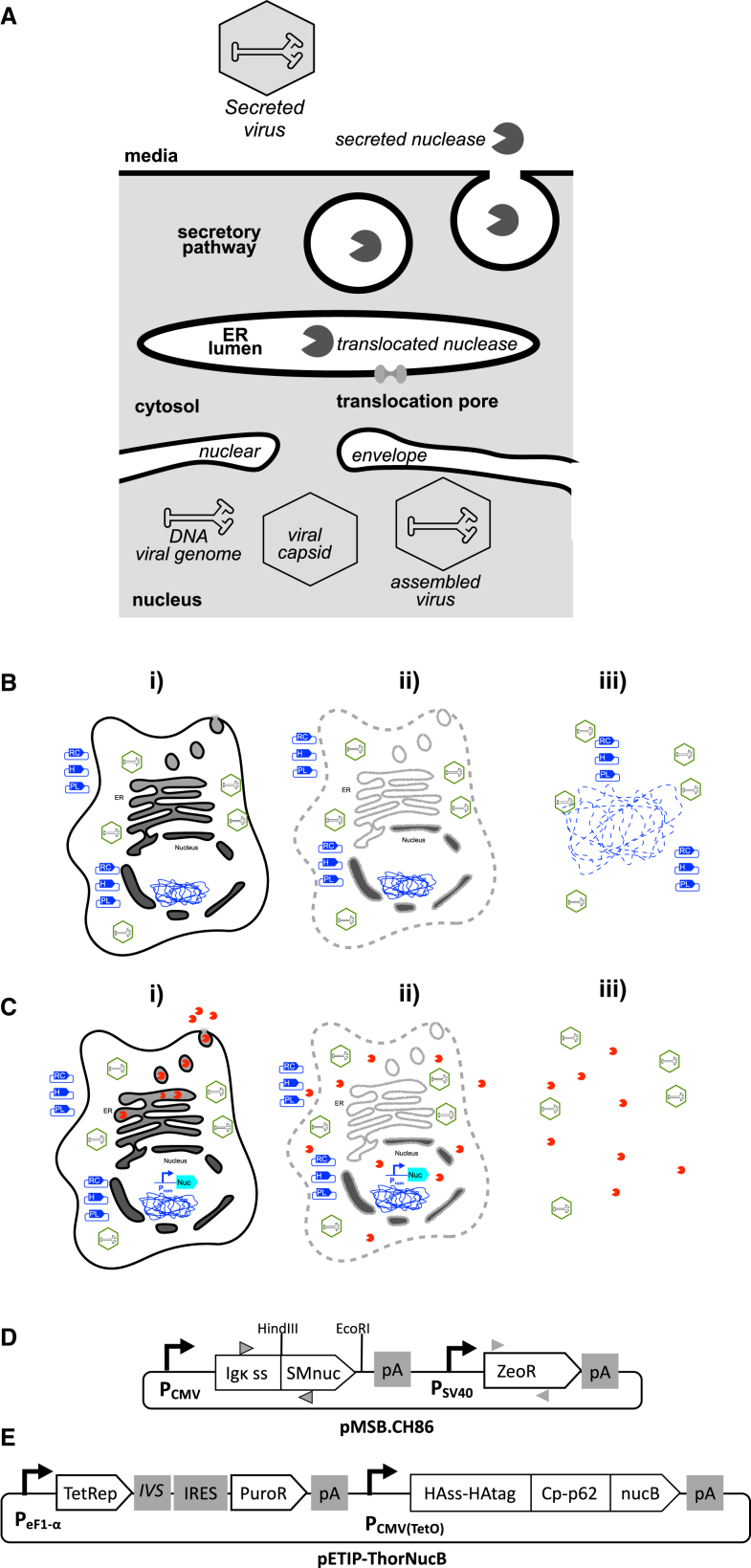
Design of plasmids and transgenes to direct secretion of bacterial nuclease from AAV packaging cells (A) Schematic overview predicting how viral genome DNA would be inaccessible to a recombinant nuclease possessing a peptide signal for its translocation to the ER lumen and ultimate transport to the cell exterior via the secretory pathway. (B) Diagram of steps in AAV production from mammalian cells: (i) transient transfection with a plasmid encoding the Rep and Cap genes (blue symbol labeled “RC”), a Helper plasmid (labeled “H”), and a plasmid encoding a recombinant “payload” genome (“PL”) brings about synthesis of progeny virus (green hexagons) by host cells; (ii) at an optimal time point cells are lysed, indicated by the dashed and fuzzy gray outlines of the cell graphic, to release progeny intracellular virus particles; (iii) centrifugation will remove cell debris, such as membrane fragments, from the process stream but not soluble nucleic acids, such as plasmid DNA and genomic DNA fragments, which may remain associated with progeny virus. (C) Anticipated AAV production from mammalian cells modified to secrete active, recombinant nuclease, indicated by the blue gene cartoon with promoter (Prom) and nuclease ORF (Nuc). (i) Transient transfection with “RC” (RepCap), “H” (Helper), and “PL” (Payload) plasmids brings about synthesis of progeny virus because recombinant nuclease is sequestered within the lumen of the vesicles and organelles of the secretory pathway, with no access to host cell nucleic acids; (ii) upon cell lysis, progeny intracellular virus particles are released and viral genomes are sequestered from recombinant nuclease by dint of being enclosed within the capsid structure; (iii) direct access allows recombinant nuclease to act upon soluble nucleic acids, such as plasmid DNA and genomic DNA fragments, reducing their presence as process impurities. (D) The pMSB.CH86 plasmid with gray triangles indicating forward (right-pointing) and reverse (left-pointing) primers used for PCR to confirm transcription of nuclease (triangles with black boundary) and zeomycin resistance (triangles without boundary). (E) The pETIP-ThorNucB plasmid encodes a “ThorNucB” open reading frame (ORF) which comprises the *S. aureus* nucB with its native secretion signal swapped for the recombinant “Thor” domain. The Thor domain consists of the influenza hemagglutinin secretion signal (HAss) and epitope tag (HAtag) fused to the carboxy-terminal domain, Cp, of the Semliki Forest virus capsid p62 protein (HAss-HAtag Cp-p62). A constitutive eF1-α housekeeping promoter is positioned upstream of an ORF for the Tet repressor (TetRep) connected to an ORF for puromycin resistance (PuroR) by a synthetic intron (IVS) and an internal ribosome entry site (IRES), to couple TetRep and PuroR marker expression. A second expression cassette features the strong, constitutive CMV promoter with a tet operator (tetO) sequence positioned immediately downstream of the TetRep binding site and upstream of the ThorNucB ORF. Polyadenylation signals are indicated by “pA” in both plasmid diagrams.

Our concept for engineering a host cell for production of AAV5 and AAV9 was firstly based on the general understanding that AAV genome replication and capsid assembly takes place outside of the interior of the organelles of the secretory pathway, principally the endoplasmic reticulum (ER), Golgi complex, and secretory vesicles. Figure 1A provides a graphically brief illustration of the predicted orthogonality of a secreted, recombinant nuclease and AAV assembly and egress phenomena.^28^ In conventional AAV harvest procedures, transient transfection with three plasmids is followed by a cell disruption step that releases progeny virus, co-releasing any nucleic acids present in the host cell (Figure 1B). We anticipate that, when the same process is applied to a nuclease-engineered host cell, cell lysis will co-release progeny virus, nucleic acids, and a recombinant nuclease that can degrade the released nucleic acids (Figure 1C). To realize this, we designed and assembled two plasmids: pSMB.CH86, encoding a serratial nuclease, SMnuc, fused to a mammalian secretion signal (Figure 1D), and pETIP-ThorNucB, encoding a staphylococcal nuclease, nucB, fused to a multicomponent secretion signal (Figure 1E).

### Nuclease activity in serum and mammalian culture media

**Figure 2:**
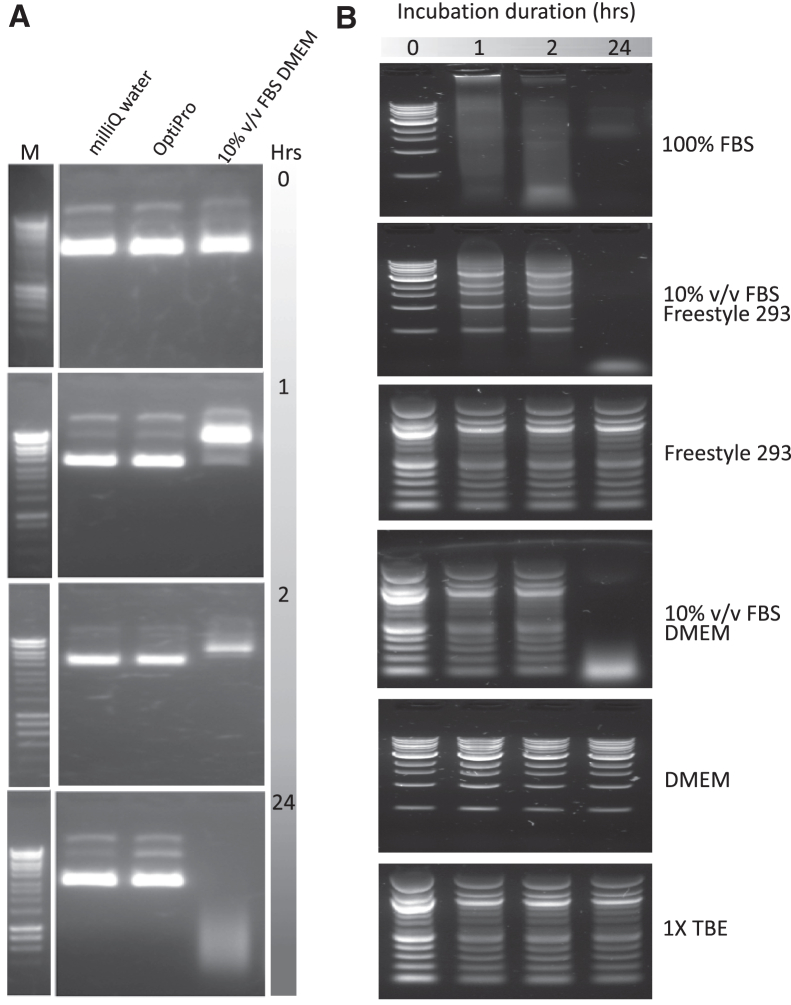
Nuclease activity in growth media (A) One microgram of the 8.8 kb plasmid, pMMB66EH, was incubated with either Milli-Q water, serum-free OptiPro medium, or DMEM with 10% v/v FBS, as described in materials and methods. These plasmid incubations were performed and ran on the lanes indicated in the uppermost image of an agarose gel stained with ethidium bromide, and in the same lane order for each gel image below. The duration of the plasmid incubations is indicated to the right of each gel image. A comparator DNA ladder, labeled “M” in the uppermost gel image, was run on the same gel as the indicated set of plasmid incubations, with non-relevant intervening lanes removed by image cropping. Uncropped images available on request. (B) Of the 1 kb New England Biolabs DNA Ladder, 1.5 μg was incubated with a range of different media and buffers for 0, 1, 2, and 24 h as described in materials and methods. These four durations of DNA ladder incubation were run in the lanes indicated in the uppermost agarose gel image of (B), and in the same lane order for each gel image below. The identity of the buffer or medium incubated with the DNA ladder is indicated to the right of each gel image.

We firstly sought to assess the nuclease activity of a range of media commonly used for serum-free and serum-based mammalian cell cultivation. Fetal bovine serum (FBS) (Thermo Fisher Scientific, UK) had previously been reported to exhibit 3′ nuclease activity in cell growth medium, measured by disappearance of DNA signal from gel electrophoresis images.^29^ When incubated with 1 μg of the plasmid pMMB66EH (Figure 2A), Milli-Q water and OptiPro serum-free media (Thermo Fisher Scientific) showed no effect over 24 h, whereas 10% v/v FBS DMEM caused significant band disappearance after 2 h and degradation to a smear of low-molecular-weight material after 24 h. Ten percent v/v FBS DMEM, 10% v/v FBS Freestyle 293, and serum alone all degraded 1 μg of the “1 kb” DNA Ladder product (cat. no. N0468S, New England Biolabs, UK) in 24 h (Figure 2B). Control incubations with Tris-borate-EDTA (TBE) (Thermo Fisher Scientific) alone, DMEM base medium alone, and Freestyle 293 base medium alone, showed no nuclease activity (Figure 2B). These data show that any serum-free AAV production platforms in future will be totally devoid of any nuclease activity not provided by process additions or engineering of host cells.

### Expressing serratial nuclease in mammalian cells

**Figure 3:**
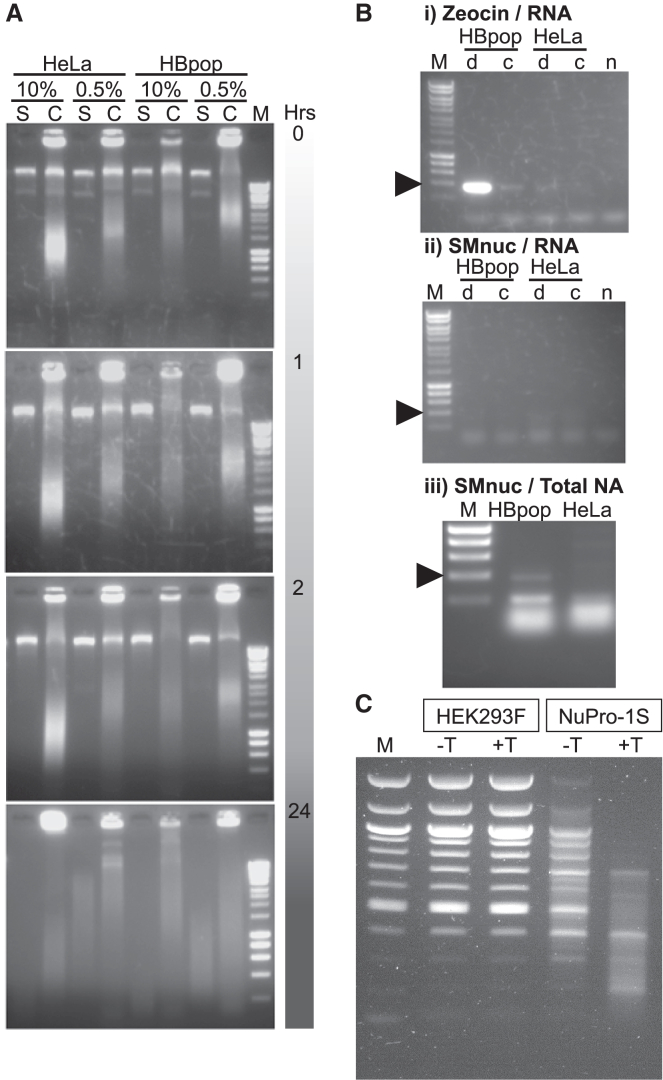
Characterizing nuclease activity arising from cells stably transfected with plasmids encoding serratial or staphylococcal nuclease Lanes labeled “M” were undigested, comparator DNA ladder throughout. (A) HeLa or HBpop cells were grown in 10% or 0.5% v/v serum growth medium for 24 h then 180 μL of growth medium, or of a solution of 1 million lysed cells, incubated with 1 μg of the plasmid pMMB66EH for 0, 1, 2, and 24 h and 80 μL of the incubation mixture ran on an agarose gel. The uppermost gel image is labeled to indicate the lanes in which pMMB66EH was incubated with growth medium (S) or lysed cells (C) of either HeLa or HBpop cultivated in serum supplemented with either 10% or 0.5% v/v serum. The lanes of all gels below were loaded in the same order with incubation duration indicated on the right. (B) (i) Total RNA extracted from HeLa and HBpop cells adapted to 0.5% v/v serum medium was reverse-transcribed to DNA (d), or used directly (c), as PCR template using primers designed to amplify a 300 bp pMSB.CH86 zeocin resistance ORF segment, with template omitted (n) as a negative control PCR. (ii) All indicated reactions are the same as (i) but primers designed to amplify a 300 bp pMSB.CH86 SMnuc ORF segment. (iii) Same PCR as (ii) performed using total nucleic acid from HBpop or HeLa cells as template. (C) Agarose gel image of 2 μL of 500 ng/μL DNA ladder (N3132S, New England Biolabs, UK) run on gel alone (lane labeled “M”), or incubated with medium arising from HEK293F cells (indicated in the labeled box above lanes) or NuPro-1S cells (likewise indicated) cultivated in the absence (–T above lane) or presence (+T above lane) of 1 μg/mL tetracycline.

We designed and assembled the plasmid, pSMB.CH86, encoding an open reading frame (ORF) for serratial nuclease, SMnuc, fused to a mammalian secretion signal (Figure 1B). We transfected HeLa cells with pSMB.CH86 to generate the stable transfectant cell line, designated “HBpop.” The initial HeLa and HBpop cell lines were both cultivated in 10% v/v FBS DMEM before subpopulations were adapted to grow in 0.5% FBS v/v DMEM. To test for nuclease activity, each of the four cell lines was passaged once overnight and the next day media taken and retained, and cells lysed. Media and lysate samples were incubated with 1 μg of the plasmid pMMB66EH for up to 24 h (Figure 3A). In the resulting gel images, for lanes where total cell lysate was run, bands corresponding to uncut pMMB66EH could be observed within the genomic DNA (gDNA) pattern. For all time points within the 24-h incubation, nucleic acid band and smear patterns arising from HBpop cells were identical to those arising from unmodified HeLa cells. Additional bands present due to plasmid supercoiling at time zero (Figure 3A, uppermost gel image) were absent after 1 h and smearing of plasmid and gDNA was broadly equivalent for all corresponding incubations after 24 h (Figure 3A, lowermost gel image).

We concluded that serratial nuclease activity was either not detectably contributing to the level of nuclease activity provided by 0.5% FBS v/v DMEM or was not occurring at all. To test for transcription of ORFs encoded by genomically integrated copies of pSMB.CH86, we performed reverse-transcription PCR (RT-PCR) to amplify DNA reverse-transcribed from mRNA purified from HeLa and HBpop cells adapted to growth in 0.5% FBS v/v DMEM. Primers specific for a region within the pSMB.CH86 zeocin resistance ORF (Figure 3B) yielded a band of expected size only for the HBpop cell line and not for unmodified HeLa cells (Figure 3Bi). A faint band of the correct size was also observed in a negative control sample of HBpop RNA that had not undergone reverse transcription (Figure 3Bi, lane 3 from the left). We concluded that this was due to HBpop gDNA impurity persisting in the RNA sample and providing a PCR template. A faint, pseudo-band for the zeocin resistance PCR performed on HeLa material (Figure 3Bi, lane 4 from the left) is artifactual, arising from the scored surface of the transilluminator.

Having gathered direct evidence for the presence of at least one transcriptionally active copy of the pSMB.CH86-encoded zeocin resistance transgene in HBpop cells, we performed the equivalent RT-PCR experiment with primers to detect mRNA encoding the SMnuc serratial nuclease (Figure 3B). No amplicon band of any size was observed for any reaction, with only low-molecular-weight nucleic acid or primer visible (Figure 3Bii). To confirm if genomically integrated DNA encoding the SMnuc ORF was present in HBpop cells, we performed conventional PCR using total purified nucleic acid from HBpop and unmodified HeLa cells as template (Figure 3Biii). An amplicon band of expected size was observed for total nucleic acid from HBpop cells and not unmodified HeLa cells.

We concluded from Figure 3B that expression of the transgenic SMnuc ORF in HBpop cells was repressed at the transcriptional level, unlike expression of the zeocin resistance ORF. This transcriptional downregulation may have resulted from unfolded protein response (UPR) caused by misfolding of SMnuc in the ER. Schofield et al. observed that the presence of a transgene encoding SMnuc in *E. coli* could reduce host cell growth and viability.^30^^,^^31^^,^^32^ We suggest that mutational screening of the primary amino acid sequence of SMnuc may be necessary for its expression in industrial host cells.

### Expressing staphylococcal nuclease in mammalian cells

We were concerned that mammalian secretion of bacterial nucleases may be generally hampered by the possible UPR phenomena we observed for SMnuc (Figure 3Bii). Because of this we assembled a plasmid, pETIP-ThorNucB, encoding a gene for nucB fused to a multi-component translocation domain featuring a segment of the Semliki Forest virus capsid protein, Cp (Figure 1C), reported to exhibit the fastest ever recorded rate of secretion from mammalian cells.^33^

Transient, triple plasmid transfection is a widely used and effective technique, despite the presence of nucleases in serum commonly used to supplement growth media. We sought to engineer inducible control, by tetracycline, into the nuclease expression cassette in pETIP-ThorNucB (Figure 1C). This “Tet-on” design feature anticipated a scenario in which nuclease secretion could be set at a basal level during transfection, and then induced at an optimal point post-transfection to preserve titer performance if needed.

Given the potential advantages of using suspension cells for AAV5 and AAV9 production compared with adherent cells, we decided to use pETIP-ThorNucB to stably transfect the commercially available HEK293F suspension cell line (Thermo Fisher Scientific), which are pre-adapted to the serum-free medium, Freestyle 293.^7^^,^^9^^,^^10^ We transfected Freestyle 293-adapted HEK293F cells with pETIP-ThorNucB to generate the stable transfectant cell line, designated “Nuclease Processing 1, suspension” (NuPro-1S).

To test for nuclease activity, medium from suspension HEK293F cells and NuPro-1S cells, cultured in the presence or absence of tetracycline, was taken 72 h post seeding and incubated with 1 μg of DNA ladder at 37°C for 1 h and analyzed by agarose gel electrophoresis. In the resulting gel image (Figure 3C), media from HEK293F cells did not result in any visible reduction in ladder DNA in the presence or absence of tetracycline compared with 1 μg untreated ladder. However, media from NuPro-1S cells cultivated in the absence of tetracycline significantly reduced the amount of DNA ladder present across all fragment lengths. Media from NuPro-1S cells cultivated in the presence of tetracycline caused significant disappearance of all >700 bp ladder bands and marked reduction of all <700 bp bands compared with 1 μg untreated ladder (Figure 3C).

### AAV5 production using the nuclease-engineered NuPro-1S cell line

We tested whether the secreted nuclease activity phenotype (Figure 3C) was compatible with AAV5 production, a clinically and commercially relevant serotype.^34^^,^^35^ We transiently transfected NuPro-1S, in the presence of 1 μg/mL tetracycline, and HEK293F cells in the absence of tetracycline, with the appropriate plasmids for production of AAV5 with a genomic payload encoding green fluorescent protein (GFP), and liberating progeny vector with a standard, bench-scale freeze-thaw process.

**Figure 4:**
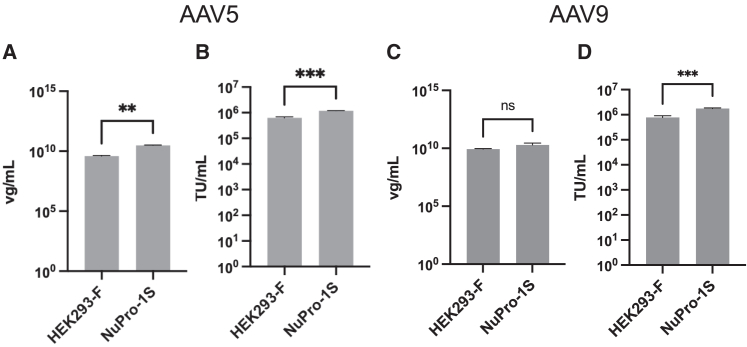
Stable transfection of HEK293F cells with a nuclease transgene does not compromise their AAV5 or AAV9 titer performance (A) Viral genomes/mL (vg/mL) as determined by qPCR. HEK293F cells yielded AAV5 particles to a titer of 3.85 × 10^9^ vg/mL, and NuPro-1S cells 3.06 × 10^10^ vg/mL. Error bars are standard deviation from *n* = 2 qPCR measurements of viral material arising from a single transfection procedure. Asterisks indicate significant difference, *p* < 0.01. (B) Transduction of adherent HEK293T cells in 10% v/v FBS DMEM, measured by flow cytometry to detect green fluorescence arising from payload genome. HEK293F cells yielded AAV5 particles to a titer of 6.27 × 10^5^ TU/mL, and NuPro-1S cells yielded AAV5 titer of 1.18 × 10^6^ TU/mL. Error bars are standard deviation from *n* = 3 flow cytometric measurements of viral material arising from a single transfection procedure. Asterisks indicate significant difference, *p* < 0.001. (C) HEK293F cells yielded AAV9 particles to a titer of 8.56 × 10^9^ vg/mL, and NuPro-1S cells 1.98 × 10^10^ vg/mL. Error bars and annotations as in (A), *p* > 0.05. (D) HEK293F cells yielded AAV9 particles to a titer of 7.77 × 10^5^ TU/mL, and NuPro-1S cells yielded AAV9 titer of 1.75 × 10^6^ TU/mL. Error bars and annotations as in (B), *p* < 0.001.

We measured the concentration of AAV5 genomes in the resultant cell lysates (Figure 4A) using a real-time quantitative PCR (qPCR) procedure that includes a nuclease treatment step to remove any potential template DNA not sequestered within intact vector vectors (materials and methods). Tet-incubated NuPro-1S cells yielded 3.06 × 10^10^ viral genomes/mL (vg/mL) while the unmodified HEK293F cells yielded 3.85 × 10^9^ vg/mL. We concluded from this that the nuclease activity secretion phenotype of NuPro-1S cells did not significantly compromise AAV5 capsid assembly and genome import.

To measure the biological activity for AAV5 viral particles arising from unmodified HEK293F and Tet-induced NuPro-1S cells we used adherent HEK293T cells as *in vitro* transduction targets.^36^ While such *in vitro* assays may not be predictive of *in vivo* function, we have used this assay to measure whether production using the NuPro-1S cell line had grossly altered infectiousness compared with unmodified host cells. NuPro-1S and HEK293F cells produced sufficient AAV5 to transduce HEK293T cells, at a level of 6.27 × 10^5^ transducing units/mL (TU/mL) and 1.18 × 10^5^ TU/mL, respectively (Figure 4B). We concluded from this that the medium-resident nuclease activity arising from NuPro-1S (Figure 3) was compatible with production of biologically active AAV5 vectors, with the same or greater yield performance as the unmodified parental HEK293F cell line.

### AAV9 production using the nuclease-engineered NuPro-1S cell line

To establish if the NuPro-1S cell line, in the presence of tetracycline inducer, could also be used to produce AAV9 vectors,^37^^,^^38^ we again used appropriate plasmids for AAV9 production with a GFP payload, liberating progeny vector with a standard bench-scale freeze-thaw process (Figure 4). As measured by qPCR, NuPro-1S produced the same or greater number of vectors as HEK293F cells, 1.98 × 10^10^ and 8.56 × 10^9^ vg/mL, respectively (Figure 4C). Biological titration with adherent HEK293T target cells also showed that NuPro-1S produced the same or greater number of transduction-competent AAV9 vectors as HEK293F cells (Figure 4D).

### Persistence of medium-resident nuclease activity from NuPro-1S cells during AAV9 processing

**Figure 5:**
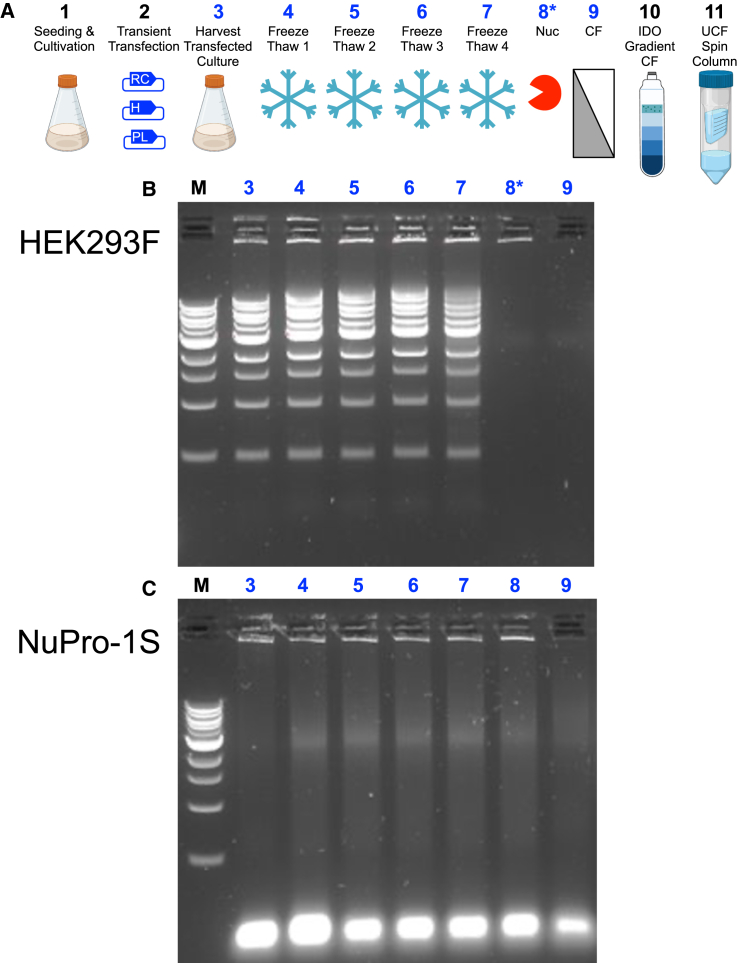
Nuclease activity in serum-free medium arising from NuPro-1S cells persists throughout an AAV9 harvest procedure (A) Graphical overview of AAV9 production and concentration procedure, as detailed in materials and methods and summarized here in 11 steps. Step 2 is transient transfection transient transfection with “RC” (RepCap), “H” (Helper), and “PL” (Payload) plasmids. When HEK293F cells are used for production, at step 8 Benzonase is added to a final concentration of 50 U/mL. When NuPro-1S cells are used no additions are made after step 7. (B) Gel image of 1 μg DNA ladder alone (lane labeled “M”) or incubated for 1 h at 37°C with 8 μL samples taken at the following steps of AAV9 harvest from HEK293F cells: step 3, cell suspension sample after transfection period complete; steps 4, 5, 6, and 7, cell suspension sample taken at the end of a thaw period; step 8, cell suspension at the end of a 1-h Benzonase incubation at 37°C; and step 9, supernatant arising from centrifugation of the cell suspension. (C) Gel image arising from the equivalent steps set out in (B), except the host cells are NuPro-1S and the step 8 incubation omitted the Benzonase addition. CF, centrifugation; UCF, ultracentrifugation; IDO, iodixanol.

We next sought to map out at which steps in the bench-scale AAV9 production process the medium-resident nuclease activity arising from NuPro-1S cells was detectable (Figure 5). The procedure we followed for bench-scale harvest and purification had 10 steps (Figure 5A), including principally a transient transfection, four freeze-thaw cycles for cell disruption, followed by centrifugation to remove cell debris prior to any further purification. Samples were taken after 7 of the 10 steps and incubated with 1 μg DNA ladder to qualitatively determine if nuclease activity was present in the sample (ladder signal disappearance) or not (ladder signal unchanged). For the parental, unmodified HEK293F cell line, no nuclease activity was detectable by this procedure for any of the tested steps in the process until step 8, the step at which commercial Benzonase was added to the process stream (Figure 5B). This nuclease activity arising from Benzonase addition persisted in the subsequent step 9; clarification by centrifugation (Figure 5B).

For NuPro-1S cells, nuclease activity was detectable in all tested steps of the process, including a modified step 8, in which no commercial Benzonase addition was made (Figure 5C). We concluded from these observations that medium-resident nuclease activity arising from NuPro-1S cells is active and persistent throughout all the principal steps of AAV9 processing.

### Reduction in levels of DNA released during cell lysis for viral vector harvest

**Figure 6:**
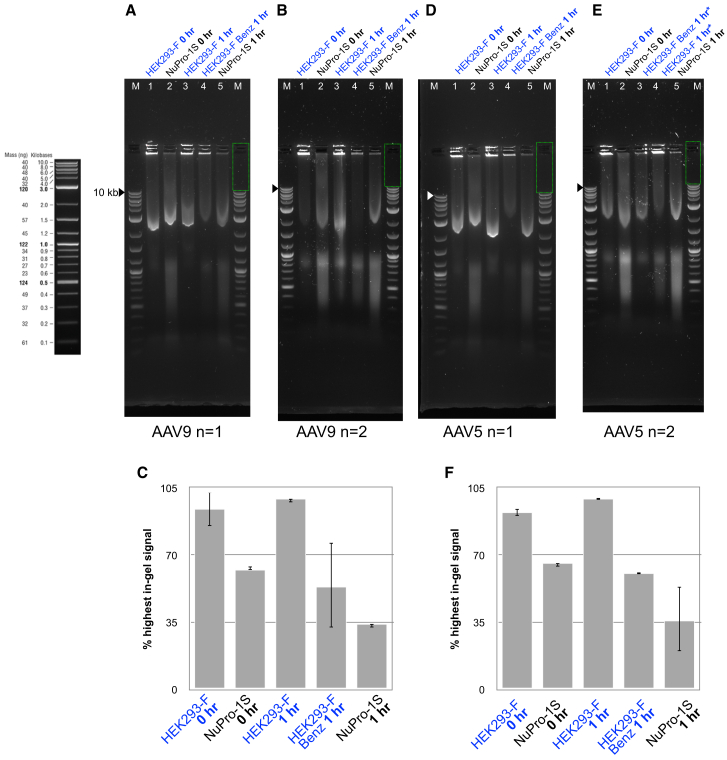
NuPro-1S cells give rise to medium-resident nuclease activity that reduces DNA impurity levels in AAV5 and AAV9 process streams HEK293F and NuPro-1S cells transfected for AAV5 and AAV9 production (titration data in Figure 4) were analyzed at step 8 (Figure 5) of the viral harvest procedure, i.e., after undergoing the fourth freeze-thaw cycle (step 7 in Figure 5) and prior to the centrifugation step for crude viral harvest (step 9 in Figure 5). (A) For AAV9, agarose gel electrophoresis was used to analyze 30 μL samples of HEK293F and NuPro-1S-derived material before (lanes 1 and 2, respectively) and after (lanes 3 and 5, respectively) a 1-h hold step at 37°C, 5% CO_2_. A further sample of HEK293F-derived material was supplemented with Benzonase, to 50 units/mL, and also subjected to the same hold step prior to gel analysis (lane 4). Nucleic acid material higher than 10 kb in length, including material retarded from migration within the sample well, was quantified using ImageJ and moving the rectangle indicated as a green dotted line on the right hand side of the gel image. The rectangle location indicated was used as the background signal for subtraction from the values for the sample lanes, 1 to 5. (B) Gel image for duplicate samples from biological repeats, with all lanes and annotation the same as the gel in (A). (C) The signal levels within the quantitation rectangle, obtained using ImageJ, were expressed as percentage of highest signal within that gel, separately for each gel. These values were then used to obtain an average and standard deviation of the two determinations (one from each gel) and plotted as indicated. (D) For AAV5, equivalent samples and gel loading were performed as in (A). (E) Gel image of duplicate samples, loaded as in (D) except for samples loaded in lanes 3 and 4, which were swapped in error (highlighted by asterisks). Quantitation in (F) is according to sample identity and not lane number. (F) Signal levels from gels in (D) and (E) were quantified as in (C). Black and white triangles indicate 10 kb bands in the ladder. Ladder key image provided on the left.

We next sought to determine the ability of the medium-resident nuclease activity arising from NuPro-1S cells (Figure 3C) to reduce the level of DNA impurities in AAV5 and AAV9 process streams. After completion of four freeze-thaw cycles (Figure 5) to liberate AAV virus, samples of lysed cells and media were analyzed by agarose gel electrophoresis either before or after a 37°C, 1-h hold step, in both the presence and absence of a Benzonase addition for HEK293F cells and with no additions for NuPro-1S cells (Figure 6A). Gel images were then analyzed via densitometry using the ImageJ software package to determine the strength of staining with the SYBR Safe DNA Gel Stain (Thermo Fisher Scientific) reagent in the migration zone for fragments of 10 kb or greater in size (Figure 6C).^39^ Notably, high levels of nucleic acid remained retarded within the sample loading well for samples from HEK293F cells, before and after the 1-h hold step (Figure 6A, compare lanes 1 and 3). We attributed this to high-molecular-weight gDNA remaining associated with cellular debris, which is consistent with other reported observations for bacterial and AAV process streams.^21^^,^^22^ When Benzonase was added to the HEK293F material for the hold step, staining in the sample well region was visibly reduced (Figure 6A, compare lanes 3 and 4). We concluded from this that the Benzonase had been active as expected and reduced the amount of high-molecular-weight nucleic acid material present.

The NuPro-1S sample taken immediately after the final freeze-thaw cycle showed a marked reduction in the amount of nucleic acid signal in the well of the agarose gel compared with the level of material in the well arising from HEK293F cells (Figure 6A, compare lanes 1 and 2). After the 37°C, 1-h hold step, nucleic acid staining for the NuPro-1S sample was reduced further compared with the post-hold HEK293F sample (Figure 6A, compare lanes 3 and 5).

Duplicate samples and procedures were performed and analyzed with a second gel (Figure 6B), which yielded comparable results. For each gel, staining of nucleic acids of 10 kb or larger was quantified as percentage of highest staining level within that gel. These relative quantitations were then averaged and plotted along with standard deviation as error bars (Figure 6C). This quantitation suggests that high-molecular-weight DNA levels are in the order of 30% lower in NuPro-1S AAV9 process streams compared with HEK293F. After a hold step, the difference in DNA impurity level widens to an approximately 60% reduction. This level of DNA reduction achieved by using the NuPro-1S cell line could also be achieved by the Benzonase addition to HEK293F material. This same overall pattern of results was also observed for AAV5 production (Figures 6D–6F).

### Transduction of cells embedded within a scaffold by AAV9 from NuPro-1S cells

We tested the ability of AAV9 vectors generated from NuPro-1S cells and regular HEK293F cells to infect cells embedded within a bioprinted scaffold. Figure S1A showed that AAV9 derived from HEK293F cells successfully transduced HEK293F cells embedded in alginate with a GFP transgene payload, resulting in cells with a green fluorescent signal that was absent from a control experiment that omitted vector (Figure S1B). Figure S1C shows that NuPro-1S-derived AAV9 vectors successfully transduced alginate-embedded target cells to a level comparable with AAV9 from the HEK293F parental cell line.

### Transduction of mouse brain tissue by concentrated AAV9 from NuPro-1S cells

We next considered whether AAV9 vectors remained associated with bacterial nuclease protein or bacterial nuclease-encoding gDNA after their production in NuPro-1S cells and subsequent concentration by ultracentrifugation. The presence of such product-related impurities may trigger innate immune responses in human patients,^16^ and hence limit the utility of the NuPro-1S cell line. As a preliminary test of this, we determined the ability of HEK293F-derived and NuPro-1S-derived, purified AAV9 vectors to transduce mouse brain tissues *in vivo* (Figure S2).

We first determined the number of genomes/mL for AAV9 derived from NuPro-1S cells and HEK293F cells after concentration by ultracentrifugation (Figure S2A). We used these concentrated samples for bilateral intracerebroventricular (ICV) injection of neonatal mice (Figure S2B). Seven days after injection with vector, brain tissue from animals injected with a vector-free negative control demonstrated zero fluorescent signal (Figure S2C), whereas tissue injected AAV9 derived from NuPro-1S cells (Figure S2D) and HEK293F cells (Figure S2E) showed highly comparable levels of green fluorescent signal.

### Long-term stability of phenotype leading to medium-resident nuclease activity

**Figure 7:**
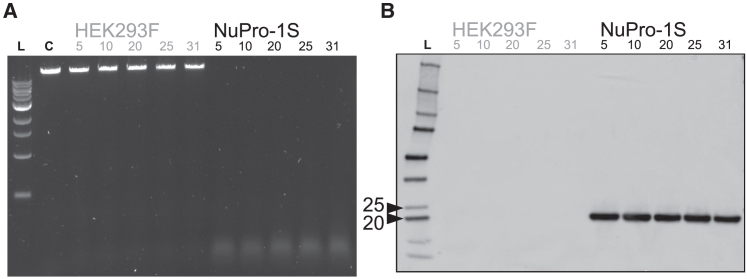
NuPro-1S phenotype that leads to medium-resident nuclease activity is stable over 31 passages (A) Agarose gel image of (lane L) DNA ladder (cat. no. N3232S, New England Biolabs), 2 μL of uncut 100 ng/μL λ DNA standard (Thermo Fisher Scientific, P11496C) alone (lane C, control) and after incubation with growth medium from HEK293F cells or NuPro-1S cells at increasing passage numbers, indicated above each lane. NuPro-1S cells were cultured in the presence of tetracycline. (B) Western blot of 5 μL of Precision plus (lane L) protein ladder (161-0376, Bio-Rad, UK) and 10 μL media samples from unmodified HEK293F cells or NuPro-1S cells at increasing passage numbers, indicated above each lane. Blot was probed with anti-HA-tag antibody. Numbered black triangles indicate the 20 and 25 kDa bands of the Precision plus ladder.

For NuPro-1S cells that had undergone a maximum of five passages, Figures 3C, 5, and 6 demonstrate a medium-resident nuclease activity that was not observed for equivalent HEK293F cells. We next tested whether the phenotype that led to this nuclease activity was stable over an extended number of up to 31 passages, performed over a 3-month period. After 10, 20, 25, and 31 passages we took media samples and incubated them with high-molecular-weight λ phage DNA (Thermo Fisher Scientific) for both HEK293F and NuPro-1S cells (Figure 7A). Incubation with media from HEK293F cells resulted in no visible degradation of the singlet λ phage DNA band for the entire duration (Figure 3A, compare HEK293F lanes with control lane). Equivalent media samples from NuPro-1S cells all caused highly similar levels of degradation of the >10 kb singlet λ phage DNA band down to a disperse smear at a <0.5 kb migration distance.

Additional, equivalent media samples from HEK293F and NuPro-1S cells were also analyzed by western blotting using an anti-hemagglutinin (HA)-tag antibody (Figure 7B). All media samples from HEK293F cells showed a total absence of antibody binding. All media samples from NuPro-1S cells showed antibody binding only to a band of between 20 and 25 kDa in size, of equivalent intensity across the entire duration of passaging.

The “ThorNucB” ORF (Figure 1) encodes a 48.42 kDa protein, consisting of, from the amino-terminal, the HA translocation signal, the HA-tag, the Semliki Forest virus capsid p62 protein, and the nucB staphylococcal nuclease. The p62 amino acid sequence features the cleavage motif, RHRR, for the Golgi-resident enzyme, furin. Post-translocational removal of the HA secretion signal, while retaining the HA-tag, followed by cleavage at the furin site, in the Golgi, would be predicted to result in a 25 kDa, HA-tag-positive protein. Pragmatic application of Occam’s razor would lead us to conclude that the ThorNucB mature protein is translocated to the ER lumen, wherein the HA translocation signal is cleaved, followed by cleavage at the furin site upon trafficking to the Golgi. The cleaved nucB nuclease and HA-tag-positive cleavage product are then co-secreted to the cell exterior.

Figure 7B does not rule out alternative scenarios, such as a proportion of the complete, uncleaved ThorNucB protein, and/or its cleavage products, being retained intracellularly and degraded, or being mis-trafficked to the cytosol and persisting for extended periods. A far more speculative possibility, not supported by any other reported observations, would be that the Semliki Forest virus capsid p62 protein possesses nuclease activity. However, all alternative scenarios must also be compatible with our observation of a medium-resident nuclease activity specific to NuPro-1S cells, which is observed to be stable over 31 passages and exerts no deleterious effect on cell growth. Further investigation into the intracellular trafficking of ThorNucB, beyond the scope of this study, will likely inform subsequent rounds of engineering of the NuPro-1S cell line to augment its effectiveness as an autonucleolytic AAV production platform.

## Discussion

Current manufacturing of AAV5 and AAV9 at scale incurs significant costs due to the need to purchase commercial nucleases that meet the quality grade required for compliance with current good manufacturing and clinical practices.^40^ Additional labor costs arise from the need to introduce this purchased nuclease material into the process stream as a process addition, and to demonstrate its removal from the product stream.^40^ Future manufacturing of AAV5 and AAV9 may look very different from the manufacturing of today, with rapidly deployable, remotely managed, modular manufacturing enabled by industrial digital technologies for bioprocesses that are fully automated from end-to-end, and that use only animal-free, chemically defined components.^13^^,^^14^^,^^15^

To help meet these current and future challenges, we have developed the NuPro-1S cell line and shown it to be capable of secreting nuclease activity to its surrounding media in a manner that persisted throughout successful transient transfection for AAV5 and AAV9 production, resulting in a reduction in DNA impurities present after cells had been disrupted to liberate progeny virus. The infectiousness of the resultant AAV5 and AAV9 material was unaffected by their production in nuclease-secreting host cells, by the measures we performed.

Future work, beyond the scope of this study, will be needed to establish if the ability of the NuPro-1S cell line to preserve the yield performance and infectiousness of AAV5 and AAV9 also holds for the other AAV serotypes commonly used in gene therapy. The NuPro-1S cell line is a polyclonal population, so obtaining and screening clonally derived populations may identify clones with better performance in terms of AAV quality, AAV yield and nuclease activity.

Additional research, utilizing techniques such as PicoGreen and qPCR, will also be needed to establish a more precise picture of the rate and type of DNA removal, for instance, whether plasmid or genomic cellular DNA are more readily degraded by the secreted, recombinant nuclease. This would be particularly required should the process be translated to commercial or clinical manufacturing. For current GMP AAV processes, Benzonase removal must be demonstrated, typically by ELISA.^41^ For the NuPro-1S cell line to be deployed in its current form it is likely that equivalent steps will also be required.

Further work will also be needed to establish if the serotype-specific tropism for specific organs and tissues of the mammalian body are preserved in AAV particles produced from NuPro-1S cells. Nevertheless, a recent patent application (WO2019175600A1) by Oxford Biomedica (UK) detailed construction of mammalian cell lines that secrete nuclease and are intended for use in AAV production. We suggest this example of commercial implementation of nucleolytic packaging cells validates the approach reported here for improving current AAV5 and AAV9 bioprocessing and enabling the development of new, algorithmically controlled, automated AAV production in the near future.

## Materials and methods

### Plasmid assembly and propagation

QIAGEN (Manchester, UK) HiSpeed Maxi kits and procedures were used for all plasmid production for transfection of mammalian cells. The pMSB.CH86 plasmid encodes expression cassettes for SMnuc with a mammalian translocation signal (Igκ ss), under control of the CMV promoter and zeomycin resistance controlled by SV40 promoter. For assembly of pMSB.CH86, firstly PCR primers were used to amplify a DNA fragment encoding SMnuc (GenBank M19495), without a translocation signal, using *S. marcescens* cells as template as described by Schofield et al.^32^ Standard molecular biology techniques were then used to subclone this fragment into the commercial plasmid, pSecTag2A (Thermo Fisher Scientific), to generate pMSB.CH86 (Figure 1B). Gray triangles in Figure 1B indicate pairs of forward (right-pointing) and reverse (left-pointing) primers used for PCR to confirm the presence and transcription of recombinant nuclease (triangles with black boundary) and zeomycin resistance (triangles without boundary). Complete pMSB.CH86 sequence data available upon request. The plasmid pETIP-ThorNucB (Figure 1C) was assembled as described by Ali et al. with sequence data available as a dataset deposited on Figshare.^42^^,^^43^

For virus production by transient transfection, plasmid pAdDeltaF6 (Addgene, plasmid no. 112867) was used to encode helper function and the payload was encoded by plasmid pscAAV-GFP (Addgene, plasmid no. 32396), incorporating an enhanced GFP (eGFP) ORF. For AAV5 capsids, plasmid pAAV2/5 (Addgene, plasmid no. 104964) was used and, for AA9 capsids, plasmid pAAV2/9n (Addgene, plasmid no. 112865) was used. Plasmid pscAAV-GFP was a kind gift from John T. Gray, plasmids pAdDeltaF6 and pAAV2/9n were kind gifts from James M. Wilson and plasmids pAAV2/5 was a kind gift from Melina Fan, all obtained via Addgene.

### Nucleic acid purification and PCRs for analysis of cells and plasmids

Total RNA was isolated from mammalian cells using the RNEasy MiniKit (cat. no. 74004, QIAGEN) following the manufacturer’s instructions. Isolated RNA was stored in TE buffer (QIAGEN) at −20°C before analysis. Conversion of total RNA to DNA, via action of reverse transcriptase, was achieved using the One-Step rtPCR kit (cat. no. 210212, QIAGEN) and following the manufacturer’s instructions. For a given cell line, total nucleic acid was isolated from 1 million cells using standard phenol/ethanol extraction and resuspended in 400 μL 10 mM Tris buffer (pH 7.5). Standard PCR was performed using reverse-transcribed DNA or total nucleic acid as template and a primer pair of either CAGTTAAGTAGCACGTGTATACGCG and GAGAGCCGGATCG, to amplify a region present in pMSB.CH86 encoding SMnuc, or CCAAGTTGACCAGTGCCGTTC and CACGAAGTGCACGCAGTTG, to amplify a region present in pMSB.CH86 encoding zeomycin resistance (see Figure 1B for overview).

### Mammalian cell cultivation and adaptation to low-serum media

All cells were incubated at 37°C with 5% CO_2_ and passaged every 3–4 days. HeLa cells were sourced from a historic master cell bank available at UCL Biochemical Engineering. HeLa cells were initially maintained in Dulbecco’s modified Eagle’s medium (DMEM) supplemented with 10% v/v heat-inactivated FBS (Sigma-Aldrich, UK). HeLa cells were adapted to grow in lower percentages of heat-inactivated FBS by serial passaging through stepwise 1% v/v decreases in FBS, with a final overall serum percentage of 0.5% v/v being achieved. Suspension-adapted HEK293F cells (cat. no. R79007) derived from HEK293 cells were purchased from Thermo Fisher Scientific and maintained in Freestyle 293 medium (Thermo Fisher Scientific). Adherent HEK293T cells were obtained from the American Type Culture Collection (cat. no. CRL-3216) and cultivated in DMEM (Gibco, UK) supplemented with 10% heat inactivated FBS (Gibco). HEK293T cells were typically seeded in wells of a Nunc Cell-Culture Treated 96-well plate (Thermo Fisher Scientific) at a viable cell density of 6 × 10^4^ cells/well.

### Mammalian cell stable transfection

Stable HeLa cell transfections were performed using 10 μg of plasmid and SuperFect reagent (QIAGEN), as per the manufacturer’s instructions, followed by selection using 0.1 μg/mL puromycin or 150 μg/mL zeocin 48 h post transfection. Transient HeLa transfections, to assess the activity of plasmid-encoded nucleases, were also performed using 10 μg of plasmid and SuperFect reagent (QIAGEN), as per the manufacturer’s instructions, but without any subsequent selection.

To generate the NuPro-1S cell line, HEK293F cells were seeded at 1 × 10^6^ cells/mL, typically in a culture volume of 10 mL, in 125 mL vent cap cell culture flasks (Corning, UK). Immediately post seeding 20 μg of plasmid DNA and the Fugene6 transfection reagent (Promega, UK) was added to cells as per the manufacturer’s protocol. The cells were cultured for 4 days post-transfection before antibiotic selection was applied using 3 μg/mL puromycin dihydrochloride (Thermo Fisher Scientific). The cells were cultured for a further 21 days under puromycin selection before verifying nucleolytic activity to be present in the cell culture medium.

### Qualitative assessment of nuclease activity

Nuclease activity in media was qualitatively assessed by adding 8 μL of a given experimental sample to (Figure 2A) a solution containing 1 μg of the 8.8 kb plasmid, pMMB66EH^44^ in nuclease-free water (Invitrogen) or (Figure 2B, 3C, and 5) 5 μL of a 500 ng/μL solution of DNA ladder (cat. no. N3232S, New England Biolabs) or (Figure 7) 2 μL of a 100 ng/μL solution of λ DNA (a component of the Thermo Fisher Scientific Quant-iT PicoGreen kit, cat. no. P11496C). All these solutions were then incubated at 37°C for the indicated durations. At the end of the incubation period, 10 μL of 0.5 M ethylenediaminetetraacetic acid (EDTA) (Invitrogen) was added to quench the reaction by sequestering the calcium ions necessary for nuclease activity. All samples were analyzed by electrophoresis through 1% w/v agarose gels buffered with TBE (Thermo Fisher Scientific) and stained with SYBR Safe DNA Gel Stain (Thermo Fisher Scientific).

Cell lysate solutions (Figure 6), in 30 μL samples, were analyzed by 1% w/v agarose gels as above. All samples were taken from procedures which used the same cells/mL cell solutions, so were considered matched at loading with respect to cells/mL prior to freeze-thaw lysis steps. Original, uncropped gel images are available on request.

### Vector production by transient transfection of cells in suspension

AAV vectors were produced in suspension cell culture by transient transfection using a process adapted from Zhao et al. and graphically summarized in Figure 5A.^9^ Suspension cells were seeded at 1 × 10^6^ cells/mL in a culture volume of 30 mL in 125 mL vent cap cell culture flasks (Corning). The cell culture was transfected immediately post-seeding using PEIpro transfection reagent (ref. no. 101000017; Polyplus Transfection, France) with helper plasmid pAdDeltaF6, payload plasmid pscAAV-GFP, and either pAAV2/5 or pAAV2/9n plasmid encoding capsid serotype 5 or and capsid serotype 9 assembly. Triple plasmid DNA mixtures were made up at a molar ratio of 1:1:1 and then added to PEIpro solution at a 1:1 volumetric ratio. The transfection mixture was added to growth media to give a final plasmid DNA concentration of 1.5 μg/mL.

Seventy-two hours after addition of transfection solution, the entire cell culture was transferred to a sterile 50 mL tube and directly subjected to four cycles of freeze-thaw using a dry ice and 70% ethanol bath. Typically 10 min duration was used for the thaw period and 15 min duration for the freeze period. Immediately after the freeze-thaw procedure, cell lysates were transferred into 125 mL shake flasks and incubated at 37°C for 1 h at 135 rpm in the presence or absence of an addition of 7.2 μL volume of Benzonase (Merck Life Science, UK) solution, to a final concentration of 50 units/mL (U/mL). Cell lysates were then clarified by centrifugation at 4,000 rpm for 30 min in a bench-top centrifuge (Centrifuge 5920 R with swinging bucket rotor S-4x1000; Eppendorf, UK) and supernatant removed via careful decanting, taking care not to disrupt the debris pellet. Supernatant was then transferred to a sterile 50 mL tube, then further aliquoted across sterile 1 and 15 mL tubes, which were then stored in a −80°C freezer (CryoCube F570n, Eppendorf) prior to any further manipulation.

To purify virus particles, clarified supernatant samples were thawed from −80°C storage and subjected to ultracentrifugation in pre-prepared iodixanol gradients (OptiPrep, Merck Life Science) at 32,000 rpm for 5.15 h at 18°C in a Beckman XPN-80 device (Beckman Coulter, Brea CA). Virus particles were resuspended in PBS-MK 0.001% v/v Pluronic-F68 (Sigma-Aldrich) prior to concentration to a suitable volume using a 100 kDa cutoff Vivaspin 20 column (Sartorius Stedim Biotech, Germany).

### AAV physical titration by qPCR with SYBR Green

Real-time qPCR was used to measure AAV payload genomes per mL of a given unconcentrated sample using a CFx Connect device (Bio-Rad, UK). The primer pair, GTCCGCCCTGAGCAAAGA and TCCAGCAGGACCATGTGATC,^45^ was used to amplify a target within the genomic payload GFP ORF, using the SsoAdvanced Universal SYBR Green Supermix (Bio-Rad) according to the manufacturer’s instructions. To remove non-encapsidated DNA prior to qPCR, a given 5 μL sample was diluted by addition of 39 μL of molecular biology-grade water (cat. no. 46-000-CM, Corning), 10 μL of 10X DNAse I reaction buffer (New England Biolabs) and 1 μL of 2 U/μL DNAseI (New England Biolabs) followed by 30 min incubation at 37°C. Samples were then incubated at 70°C for 10 min to denature capsids such that they released their payload genome contents. Serial dilution was then performed before qPCR. Dilutions of the pscAAV.GFP plasmid of known purity and concentration were used to establish a standard curve of cycle threshold as a function of template mass. For concentrated samples, the same procedure as above was performed, except with a StepOne Real-Time PCR System device (Applied Biosystems) and SYBR Green Master Mix (Bio-Rad) using the following primer pair to amplify a region within the inverted terminal repeat region: GGAACCCCTAGTGATGGAGTT and CGGCCTCAGTGAGCGA.^46^

### Determination of AAV biological activity by transduction of target cells

Biological activity of AAV viral particles was quantified by measuring their ability to transduce adherent HEK293T cells. Samples containing viral particles were thawed on ice prior to preparing serial dilutions ranging from neat to 1:125,000 in DMEM supplemented with 10% v/v FBS. Twenty microliters of neat or diluted viral vector was then added to wells of a 96-well plate, containing HEK293T cells at 70% confluence. Seventy-two hours post transduction, transduced cells were harvested by trypsinization and fixed using 4% v/v paraformaldehyde solution in phosphate-buffered saline (PBS) (Alfa Aesar, UK) and fluorescence measured by flow cytometry using the BD Fortes flow cytometer (BD Biosciences, UK) with excitation at 488 nm to detect GFP signal. The percentage of GFP-positive cells was measured and normalized using non-transduced cells. The number of TU/mL was calculated using Equation 1 below.

(Equation 1) TU/mL={[(%GFPpositivecells)∗(no.cellsattransduction)]/vectorinputvolume}∗dilutionactor

Data gathered from flow cytometry experiments were analyzed using Fojo v.10.7.1 (BD Biosciences) software. To count the number of GFP-positive cells, gates were applied to isolate cells, singlet cells, and GFP-positive cells. A minimum of 10,000 cell measurement events within the gate were recorded per sample and used for analysis.

### AAV9 transduction of target cells embedded in tissue mimic the scaffold

To prepare the scaffold, 2.4 g sodium alginate powder (cat. no. A2033, Merck Life Science) was mixed with 20 mL Freestyle 293 medium, containing 1% v/v antibiotic-antimycotic (10 mg/mL streptomycin, 25 μg/mL amphotericin B, and 10,000 units/mL penicillin, from Thermo Fisher Scientific), in a 100 mL Pyrex Duran bottle placed on a Corning PC-420D model hot plate set at 50°C, all within a class II Microbiological Safety Cabinet (Walker, UK). When the solution had cooled to approximately 35°C–40°C, assessed by touch, 180 μL aliquots of the alginate scaffold were each transferred to 1.5 mL Eppendorf tubes. Twenty microliters of a 1.8 × 10^7^ HEK293F cells/mL cell solution, in the same Freestyle 293 medium, was then added to a given Eppendorf tube and mixed using a P200 yellow tip pipette (cat. no. 70.760.211, Sardstedt, Germany) via repeated dispensing and withdrawing of the solution with the tip end remaining submerged throughout to avoid frothing. Each 200 μL aliquot of cell-containing scaffold assembled in this way was then transferred to a single well of a 24-well plate for setting of the scaffold to complete at room temperature. One hundred microliters of Freestyle 293 medium only, or 100 μL AAV9 solution, was added on top of the set scaffold within the well. The 24-well plate was then transferred to a Hera Cell 150 incubator (Thermo Fisher Scientific) set at 37°C, 5% CO_2_ for 24 h prior to imaging. Wells were imaged using an EVOS FL microscope (EVOS FL, Thermo Fisher Scientific) using a phase-contrast or a GFP light cube (470 nm excitation, 525 nm emission) at a 50% illumination setting at 10× magnification. An EVOS light shield box (Thermo Fisher Scientific) was placed around the 24-well plate prior to GFP imaging to minimize background fluorescence caused by alginate scaffold.

### AAV9 transduction of target cells in mice

All procedures were approved by the UK Home Office for the conduct of regulated procedures under license (UK Animal Scientific Procedure Act, 1986) and by the University College London Animal Welfare and Ethical Review Board (AWERB). The Animal Research Reporting of In Vivo Experiments (ARRIVE) guidelines from the National Center for the Replacement Refinement and Reduction of Animals in Research were followed. Animals were maintained in individually ventilated cages, on a 12-h light/dark cycle, with access to water and food *ad libitum*.

scAAV9 vectors carrying eGFP reporter gene (scAAV9.eGFP) were used to characterize *in vivo* functionality of vectors produced from HEK293F and the nuclease-engineered NuPro-1S cell line. Concentrated virus solution was diluted to 7 × 10^12^ vg/mL with PBS prior to administration. At day of birth (post-natal P0), CD1 strain wild-type pups were administered with 7 × 10^13^ vg/kg of each concentrated viral vector (four mice/cohort). The vector was injected via bilateral ICV injection targeting the anterior horn of the lateral ventricle using a 33-gauge needle (Hamilton, Reno, NV). Three littermates were administered with 10 μL of PBS (5 μL/hemisphere) and kept as controls. Injected pups were subsequently returned to the dam. Seven days post-administration mice were euthanized via terminal exsanguination by trans-cardiac perfusion with PBS. Brains were subsequently extracted and immediately imaged with a DFC700 T camera mounted onto a MZ16F microscope (Leica, Wetzlar, Germany).

### Western blotting

Medium was analyzed by western blotting using equal volumes of media obtained from HEK293F or NuPro-1S cells. In brief, cells were seeded at 1 × 10^6^ cells/mL in 125 mL shake flasks in Freestyle 293. No additions were made to 293F cells while 1 μg/mL of tetracycline was added to NuPro-1S cells 24 h post seeding. Medium samples were obtained by centrifuging the cell culture samples 72 h post seeding, with supernatant retained and stored at −80°C. Supernatant samples were thawed and 45 μL added to 13.5 μL 4X Laemmli buffer (Bio-Rad) and 1.5 μL β-mercaptoethanol (Bio-Rad) then incubated at 95°C for 5 min in a hot block. Ten microliters of this denatured sample was then loaded into a precast SDS-PAGE gel (no. 4561084, Bio-Rad) and ran at 150 V for 50 min. Proteins were transferred to PVDF membranes (no. 1704156, Bio-Rad) and probed using rabbit anti-HA-tag antibody (SG77, Thermo Fisher Scientific) at a 1:500 dilution followed by overnight incubation at 4°C. The membrane was then probed with HRP-conjugated goat anti-rabbit antibody (no. 31460, Thermo Fisher Scientific) at a dilution of 1:20,000 and incubated for 1 h prior to chemiluminescent imaging (no. 1705062 Clarity Max, Bio-Rad) using an Amersham Imager 600 device (Cytiva, UK).

## Data and code availability

Raw data supporting the findings of this study are available from the corresponding author on request. The sequence of pETIP-ThorNucB is available at the Figshare online dataset repository, https://doi.org/10.6084/m9.figshare.22770212.v1.

## Acknowledgments

We gratefully acknowledge the support of the UK 10.13039/501100000266Engineering and Physical Sciences Research Council (10.13039/501100000266EPSRC) via grant number EP/S021868/1. We acknowledge the support of the Republic of Turkey Ministry of National Education for support for M.B.

## Author contributions

D.N.N., G.H., J.W., and E.K.-M. conceived the study. G.H., M.B., M.W., G.M., A.A.R., A.O., D.M.S., S.A., M.R., J.W., E.K.-M., C.M., and D.N.N. contributed to design of methodology and investigation. G.H., M.B., M.W., G.M., A.A.R., A.O., D.S., S.A., M.R., and D.N.N. contributed to data visualization. G.H., M.B., M.W., G.M., A.A.R., A.O., D.S., S.A., M.R., J.W., E.K.-M., C.M., and D.N.N. supervised the project. G.H., M.B., M.W., G.M., and D.N. wrote the manuscript. D.N.N., G.H., M.B., M.W., and G.M. reviewed and edited the manuscript.

## Declaration of interests

The authors declare no competing interests.

## Supplemental information

Document S1. Figures S1 and S2

Document S2. Article plus supplemental information
