# Supplementary material for: An autonucleolytic suspension HEK293F host cell line for high-titer serum-free AAV5 and AAV9 production with reduced levels of DNA impurity
Source: Mol Ther Methods Clin Dev. 2024 Aug 12;32(3):101317. doi: 10.1016/j.omtm.2024.101317 (PMC11385518; doi:10.1016/j.omtm.2024.101317)
Supplement: Document S1. Figures S1 and S2 [file mmc1.pdf]

OMTM, Volume *32*

Supplemental information

An autonucleolytic suspension HEK293F host cell line for high-titer serum-free AAV5 and AAV9 production with reduced levels of DNA impurity

Geoffrey Howe, Mehtap Bal, Matt Wasmuth, Giulia Massaro, Ahad A. Rahim, Sadfer Ali, Milena Rivera, Desmond M. Schofield, Aminat Omotosho, John Ward, Eli Keshavarz-Moore, Chris Mason, and Darren N. Nesbeth

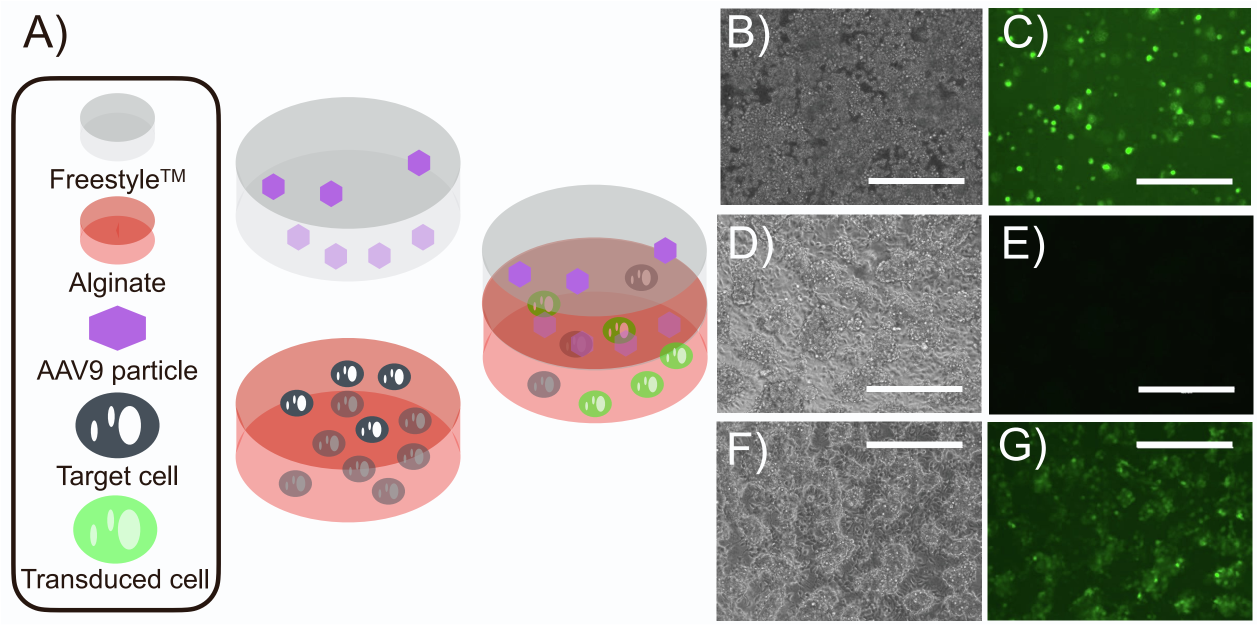

Figure S1. AAV9 produced using NuPro-1S cells can transduce target cells embedded within a scaffold tissue mimic. Phase contrast and fluorescent images of HEK293-F cells embedded within a plug of alginate scaffold onto which a solution of AAV9 vector particles in Freestyle 293™ media, from the same production runs characterised in Figure 5, was added. **A)** Schematic diagram of experimental setup with key panel on left. 100 µL scaffold with embedded cells in a well of a 24-well plate and 100 µL vector solution prepared separately (middle of diagram) and vector solution added on top of scaffold (rightmost portion of diagram) for green fluorescent signal to report transduction events. Phase contrast (**B**) and fluorescent (**C**) images taken from a well in which 100 µL AAV9 vectors derived from unmodified HEK293F cell were added to the scaffold plug. Equivalent images (**D**, **E**) were taken when 100 µL Freestyle 293™ media only were added to a scaffold plug. Corresponding images (**F**, **G**) also captured when 100 µL AAV9 derived from NuPro-1S cell were added to the scaffold plug. Scale bars indicate 400 µm.

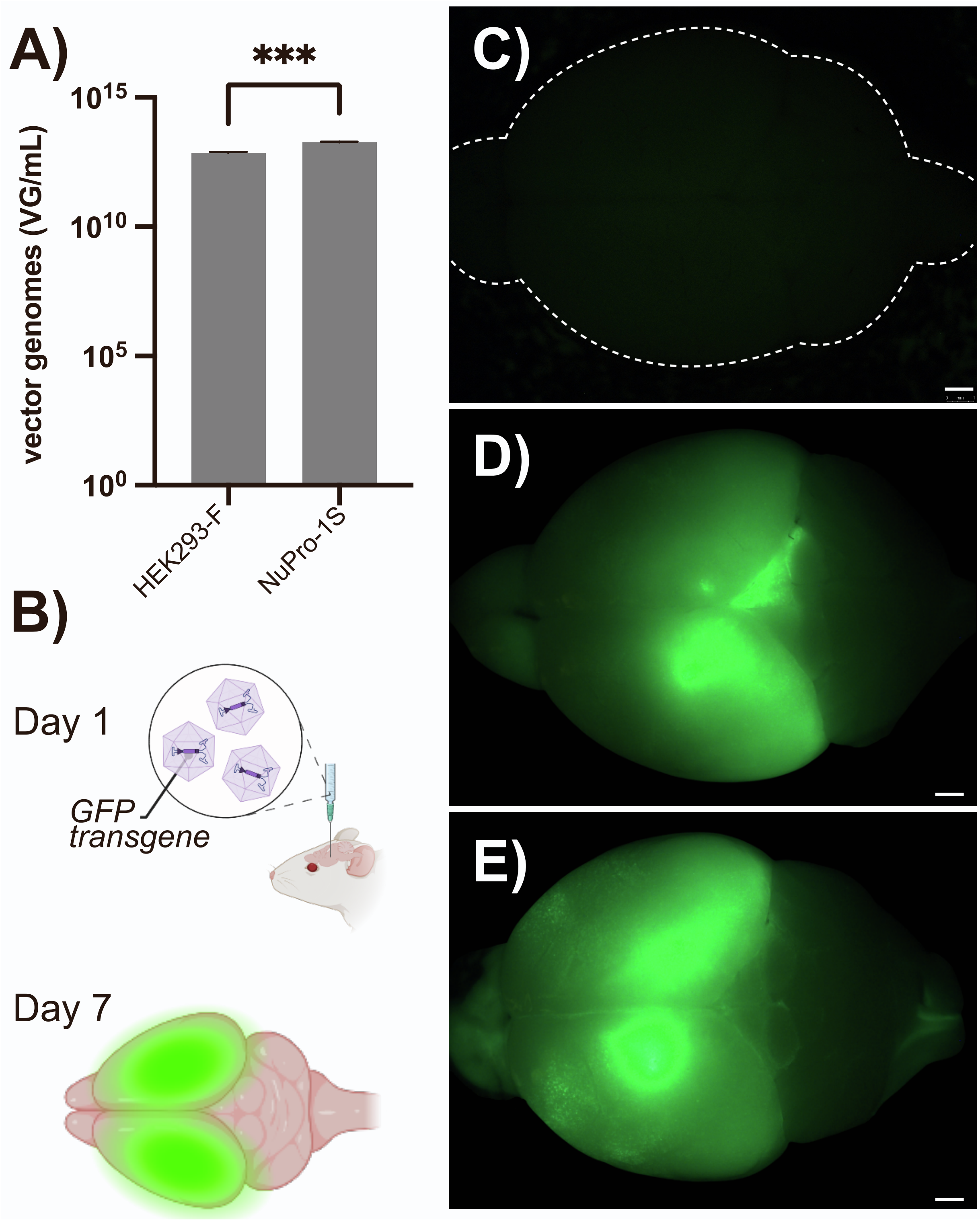

Figure S2. AAV9 produced using NuPro-1S cells can transduce target mouse neonate brain cells *in vivo*. **A**) AAV9 material previously characterised in Figure 4 was concentrated by iodixanol gradient ultracentrifugation followed by buffer exchange using a centrifugal concentrator (Figure 5 for overview) and physical titre measured by qPCR using ITR-

directed primers. Error bars are standard deviation of n=2 qPCR determinations. Data are representative of a single transfection procedure to produce virus. Asterisks indicate significant difference P < 0.001. **B**) Day 1 - Graphical depiction of concentrated vector administration intracerebrally to neonatal mice Day 7 -. Graphical depiction of resulting green fluorescence anticipated to arise in the left and right hemispheres of the injected rate brain, in the context of surrounding brain structures. **C**) Fluorescent image of extracted brain seven days after bilateral intracerebroventricular (ICV) injection of phosphate buffered saline (5 µL/hemisphere) as negative control. Dashed line indicates location of brain. **D**) Fluorescent image captured in same conditions as previous image but injection contained $7x10^{13}$ viral genomes / kg mouse body weight (vg/kg) of AAV9 derived from HEK293-F cells. **E**) Fluorescent image resulting from injection $7x10^{13}$ vg/kg of AAV9 derived from NuPro-1S cells. All fluorescent images include 1 mm scale bar in bottom right and are representative of n=4 independent viral injection procedures.
